# Energy Landscape of All-Atom Protein-Protein Interactions Revealed by Multiscale Enhanced Sampling

**DOI:** 10.1371/journal.pcbi.1003901

**Published:** 2014-10-23

**Authors:** Kei Moritsugu, Tohru Terada, Akinori Kidera

**Affiliations:** 1 Computational Science Research Program, RIKEN, Hirosawa, Wako, Saitama, Japan; 2 Graduate School of Medical Life Science, Yokohama City University, Suehiro-cho, Tsurumi-ku, Yokohama, Japan; 3 Graduate School of Agricultural and Life Sciences, The University of Tokyo, Yayoi, Bunkyo-ku, Tokyo, Japan; Baltimore, United States of America

## Abstract

Protein-protein interactions are regulated by a subtle balance of complicated atomic interactions and solvation at the interface. To understand such an elusive phenomenon, it is necessary to thoroughly survey the large configurational space from the stable complex structure to the dissociated states using the all-atom model in explicit solvent and to delineate the energy landscape of protein-protein interactions. In this study, we carried out a multiscale enhanced sampling (MSES) simulation of the formation of a barnase-barstar complex, which is a protein complex characterized by an extraordinary tight and fast binding, to determine the energy landscape of atomistic protein-protein interactions. The MSES adopts a multicopy and multiscale scheme to enable for the enhanced sampling of the all-atom model of large proteins including explicit solvent. During the 100-ns MSES simulation of the barnase-barstar system, we observed the association-dissociation processes of the atomistic protein complex in solution several times, which contained not only the native complex structure but also fully non-native configurations. The sampled distributions suggest that a large variety of non-native states went downhill to the stable complex structure, like a fast folding on a funnel-like potential. This funnel landscape is attributed to dominant configurations in the early stage of the association process characterized by near-native orientations, which will accelerate the native inter-molecular interactions. These configurations are guided mostly by the shape complementarity between barnase and barstar, and lead to the fast formation of the final complex structure along the downhill energy landscape.

## Author Summary

Dynamic nature of the protein-protein interactions is an important element of cellular processes such as metabolic reactions and signal transduction, but its atomistic details are still unclear. Computational survey using molecular dynamics simulation is a straightforward method to elucidate these atomistic protein-protein interaction processes. However, a sufficient configurational sampling of the large system containing the atomistic protein complex model and explicit solvent remains a great challenge due to the long timescale involved. Here, we demonstrate that the multiscale enhanced sampling (MSES) successfully captured the atomistic details of the association/dissociation processes of a barnase-barstar complex covering the sampled space from the native complex structure to fully non-native configurations. The landscape derived from the simulation indicates that the association process is funnel-like downhill, analogously to the funnel landscape of fast-folding proteins. The funnel was found to be originated from near-native orientations guided by the shape complementarity between barnase and barstar, accelerating the formation of native inter-molecular interactions to complete the final complex structure.

## Introduction

Protein-protein interactions are the fundamental components in the interaction networks describing cellular processes such as metabolic reactions and signal transduction. When trying to acquire a more detailed understanding of the association and dissociation processes of protein complexes, however, we encounter some complicated physics involved in these protein-protein interactions, in which a subtle balance between the weak atomic interactions and solvation determines the marginal stability/affinity and the specificity [1]–[3]. Such a physical picture is reminiscent of the complexity in protein folding, which has been overviewed from the energy landscape picture linking the unfolded states to the folded state [4], [5]. Likewise, an energy landscape of protein-protein interactions linking the dissociated states to the unique stable complex structure [6]–[8] is necessary.

There are two stages in the process involved in the formation of a protein complex, the “diffusion-collision” process from the fully separated states to the encounter complex, and the “association” process from the encounter complex to the native complex structure. The formation of the encounter complex has been experimentally well studied by using the Förster resonance energy transfer [9], [10], atomic microscopy [11]–[13], transferred NOE spectroscopy [14], [15], paramagnetic relaxation enhancement [16]–[18], and computationally by conducting Brownian dynamics simulations [19]–[25]. On the other hand, study of the second stage, which is the formation of the tightly bound native complex structure, still remains a challenge both experimentally and theoretically due to the difficulties in detecting the atomic-detailed process of the formation of complicated interactions including the desolvation at the interface of protein complexes. In particular, the large scale configurational sampling by conventional equilibrium molecular dynamics (MD) simulations is a difficult task due to the slow kinetics and a large number of degrees of freedom in the sampling space. To solve this problem more elaborate simulation techniques have been used to calculate the free energy surface (FES), such as steered MD [26], constrained MD [27], and the weighted histogram analysis method [28]. These simulations introduced a single dimensional reaction coordinate, such as the distance between the centers of mass for the two proteins, connecting the bound state and a dissociated state, to reduce the sampling space. However, the potential of mean force along a pre-fixed one-dimension is too simple for describing the FES of the complicated protein-protein interactions, just as in the protein folding problem that requires many dimensions for a proper description of the FES in the folding funnel landscape. The simplest and most direct way to solve the problem is thus a full configurational sampling of the protein-protein interactions.

In this study, we try to directly obtain the energy landscape, or the FES, of the all-atom protein-protein interactions during the association process in explicit solvent by conducting a multiscale enhanced sampling (MSES) simulation [29]–[31]. The MSES enhances the sampling by using a multiscale scheme where the all-atom model (MM) is coupled with the accelerated dynamics of the coarse-grained (CG) degrees of freedom, together with the Hamiltonian replica exchange method to eliminate the bias of the coupling to the CG model [32]–[38]. The scalability in the Hamiltonian replica exchange for application to large protein systems is attained by setting the dimensionality of the CG model small enough to represent only the “essential subspace”. Our previous studies on the folding dynamics of chignolin [29], [31] and on the ordering transition of an intrinsically disordered protein (sortase) [30] have demonstrated the outstanding capability of the all-atom conformational samplings of large proteins in explicit solvent. The use of multiscale scheme has also been aimed to develop the CG force fields from the MM simulations by bottom-up approach [32]–[34], [38], and applied for enhanced sampling such as resolution replica exchange [39], [40], adiabatic coupling [41], [42] and temperature accelerated MD [43]–[46].

We chose the barnase-barstar complex, which is a bacterial RNase bound to its inhibitor [47]–[49], as a model protein complex to study the association dynamics. This complex is characterized by its extraordinary tight binding (*K*_d_ = 10^−14^ M) [50] and fast binding kinetics (*k*_on_ = 10^8^ s^−1^M^−1^) [51]. Comparative mutation studies revealed that the fast and tight binding is due to a significant electrostatic complementarity between the two protein interfaces [50]–[54]. Brownian dynamics simulations successfully reproduced the diffusion process of the mutant complexes under various environmental conditions [19]–[25]. Here, the FES of the barnase-barstar interaction during the association process after the encounter was calculated using the MSES simulation to investigate how the electrostatic and shape complementarity determined the energy landscape for the processes of the formation of the native intermolecular contacts and desolvation of the hydrated waters.

## Results

### MSES simulation

**Figure 1:**
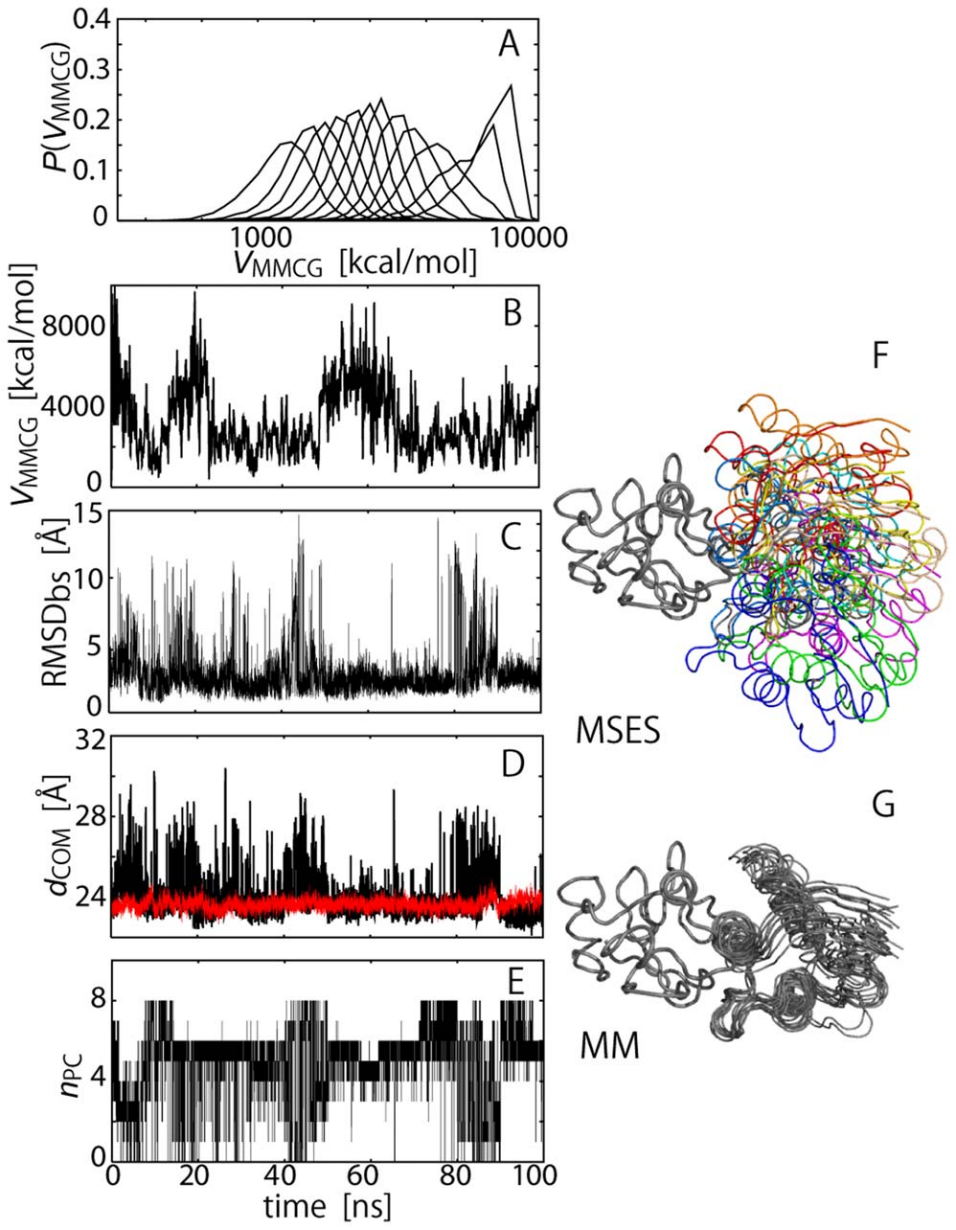
MSES simulation. (A) Probability distributions of *V*_MMCG_, *P*(*V*_MMCG_), defined in Eq. 1 for 12 replicas of MSES simulation. (B) Time course of *V*_MMCG_ for a representative model replica, i.e., the replica fixed not by *k*_MMCG_, but by the configuration. (C–E) Quantities from the unbiased MSES ensemble (with *k*_MMCG_ = 0) as a function of simulation time: Root-mean-square displacement for C_α_ atoms (C_α_ RMSD) of barstar after fitting to barnase, RMSD_bs_ (C), center-of-mass (COM) distance between two COMs for barstar and barnase, respectively, *d*_COM_ (D), and number of polar contacts found in eight inter-molecular pairs, #3, 4, 6, 7, 8, 11, 12, and 13, listed in Table 1 (E). In (D), *d*_COM_ in conventional equilibrium MD simulation starting from complex structure (MM simulation) is also shown by red. In (F) and (G), arrangements of barstar observed in the unbiased MSES ensemble and in MM simulation are shown, respectively. Both coordinates were superimposed on barnase.

The MSES simulation of barnase and barstar in explicit solvent was performed to fully sample the all-atom configurations during the association process to form the native complex structure. Twelve replicas for the Hamiltonian exchange were sufficient for simulating the solvated system containing ∼35,000 atoms, owing to the high scalability of the MSES [29]–[31]. The energy distribution of the MM/CG coupling term (see Eq. 1 in Methods) significantly overlaps the distributions of the neighboring replicas (Fig. 1A), guaranteeing a high exchange probability or a successful Hamiltonian exchange simulation; the average acceptance ratio of the exchange was 0.25. The fluctuation of *V*_MMCG_ in Eq. 2 shows sufficient swapping of *k*_MMCG_ in all the replicas, indicating the successful simulation of the Hamiltonian replica exchange (Fig. 1B and Fig. S1).

The enhanced sampling of the barnase-barstar system was achieved by using the following MSES procedure. The potential energy of the CG model was set as the Lennar-Jones type potential for the protein-protein interactions, which has a shallow minimum for the native complex structure and a broad potential of non-native states (see Methods for details). This CG potential energy plays a role in leading the barstar to frequently move back and forth between the bound and unbound state, rather than favoring the bound state, as indicated in the FES exhibiting a single minimum at the intermediate distance (Fig. S2). The strong coupling with the CG models (a large value of the coupling constant, *k*_MMCG_, see Eq. 1 in Methods) drives the MM model to sample a large configurational space to provide a broad distribution, as shown in the FES for all the replicas showing close similarity to that for the CG force field (Fig. S2). We obtained the FES of the unbiased potential (*V*_MM_ in Eq. 1) by extrapolating *k*_MMCG_ to zero, which is depicted as the configurational ensemble covering a much larger configurational space than that sampled by the conventional equilibrium MD simulation (Fig. 1F and 1G, respectively; hereafter we call the latter the MM simulation).

The MSES ensemble for the unbiased simulation with *k*_MMCG_ = 0 shows that the barnase and barstar molecules experience the association and dissociation processes several times, thus traversing a large configurational space (Figs. 1C–E), which is seen in RMSD_bs_ (C_α_ root-mean-square displacement (RMSD) of barstar after superimposing barnase) = 1–15 Å, *d*_COM_ (the center-of-mass (COM) distance between the two proteins) = 22–30 Å (*d*_COM_ = 23.2 Å for the complex structure in the Protein Data Bank (PDB):1BRS [49] and *d*_COM_<25 Å for the MM simulation), the number of inter-molecular polar contacts (out of the eight contacts listed in Table 1) = 0–8, and in representative structures (Fig. 1F). The proteins in all the replicas maintained their stability during the MSES simulation (C_α_ RMSD within barnase and barstar being <1.5 and 1.3 Å, respectively, for any replica with a finite value of *k*_MMCG_). The configurational sampling of the protein-protein interaction process when using the all-atom model in explicit solvent allows for a straightforward analysis of the energy landscape.

**Table 1:** Probability of polar contact formation.

#^a^	barnase	barstar	*p*_MM_ b	*p*_MSES_ c_ Cα RMSD<4 Å_
1	Lys27N_ζ_	Thr42O_γ_	0.21	0.51
2	Ser38N	Trp44O	0.02	0.15
3	Arg59N	Asp35O_δ_	**1.00**	**0.71**
4	Arg59N_η_	Glu76O_ε_	**0.99**	**0.79**
5	Glu60N	Asp35O_δ_	0.34	0.07
6	Glu60O_ε_	Leu34N	**0.72**	**0.56**
7	Arg83O	Tyr29O_η_	**0.85**	**0.69**
8	Arg83N_η_	Asp39O_δ_	**1.00**	**0.80**
9	Arg83N_η_	Gly43O	**0.96**	0.41
10	Asn84O	Tyr29O_η_	0.00	0.29
11	Arg87N_η_	Asp39O_δ_	**1.00**	**0.78**
12	His102O	Asn33N_δ_	**0.99**	**0.95**
13	His102N_ε2_	Asp39O_δ_	**0.99**	**0.93**
14	Tyr103O	Asn33N_δ_	0.18	0.24
15	Tyr103O_η_	Asp39O_δ_	0.58	0.48

a List was made according to polar contacts formed in the complex crystal structures [54]. The bold numbers indicate that *p*_MM_>0.70.

b *p*_MM_ for probability in MM simulation starting from the complex structure.

c *p*_MSES_ for probability in MSES simulation where C_α_ RMSD<4 Å from the complex structure.

We analyzed the polar contact network at the interface to examine the protein-protein interactions at the atomistic resolution. Fifteen inter-molecular polar contacts formed in the crystal structures [54] were chosen to calculate the contact probability in the MSES ensemble (Table 1). It is demonstrated in Table 1 that the probability of forming the polar contacts observed in the near-native structures of the MSES ensemble (C_α_ RMSD of barstar after superimposing barnase is less than 4 Å) have almost the same pattern of the probability observed in the MM simulation that started from the crystal structure (Table 1); the correlation coefficient between the two columns was 0.83. This indicates that the atomistic interactions on the interface were correctly reproduced during the large-scale association-dissociation process in the MSES simulation.

### Funnel-like downhill energy landscape of protein-protein interactions

**Figure 2:**
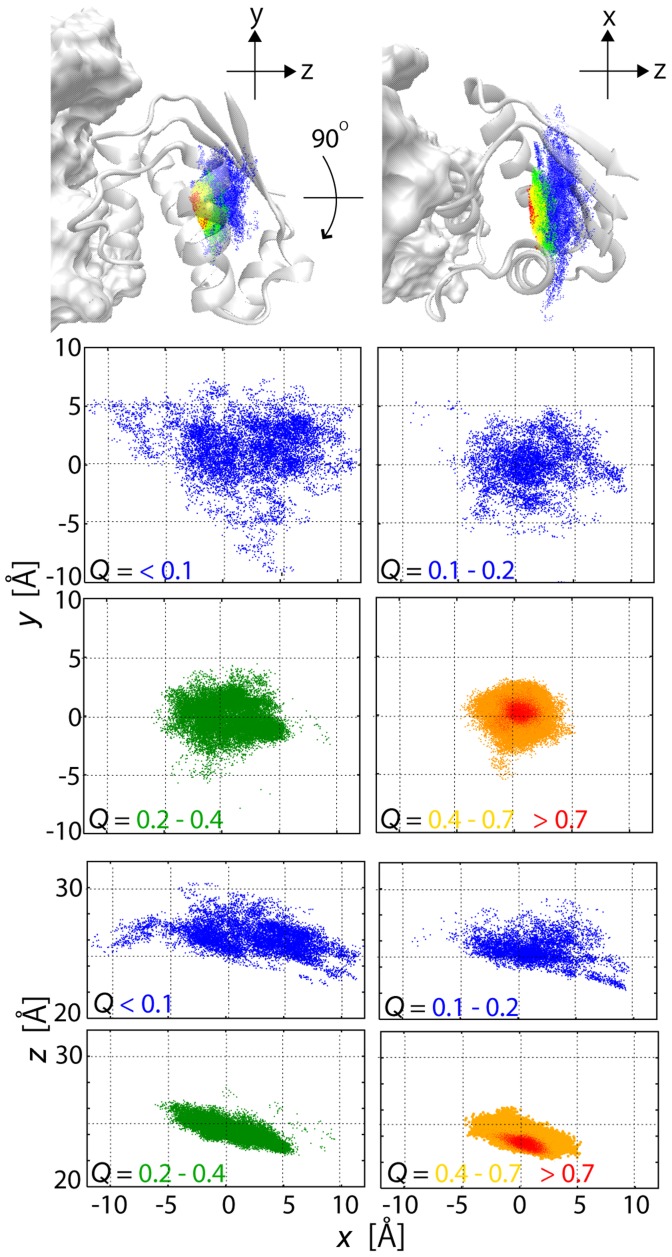
Funnel landscape of barnase-barstar interaction. Distributions of centers of mass (COM) of barstar with various ranges of fraction of native inter-molecular contacts formed (*Q*) after superimposing barnase in unbiased ensemble of MSES simulation. (Top) Three-dimensional distributions at *Q*<0.2 (blue), 0.2<*Q*<0.4 (green), 0.4<*Q*<0.7 (yellow), and *Q*>0.7 (red). (Bottom) Distributions onto *x-y* plane and *x-z* plane at depicted *Q* ranges. The *x*-*y* plane was defined to be orthogonal to the vector connecting the two COM's of barnase and barstar (*z*-axis), and *x*-axis being the direction of the vector from C_α_ of Arg87 to C_α_ of Arg83 of barnase.

**Figure 3:**
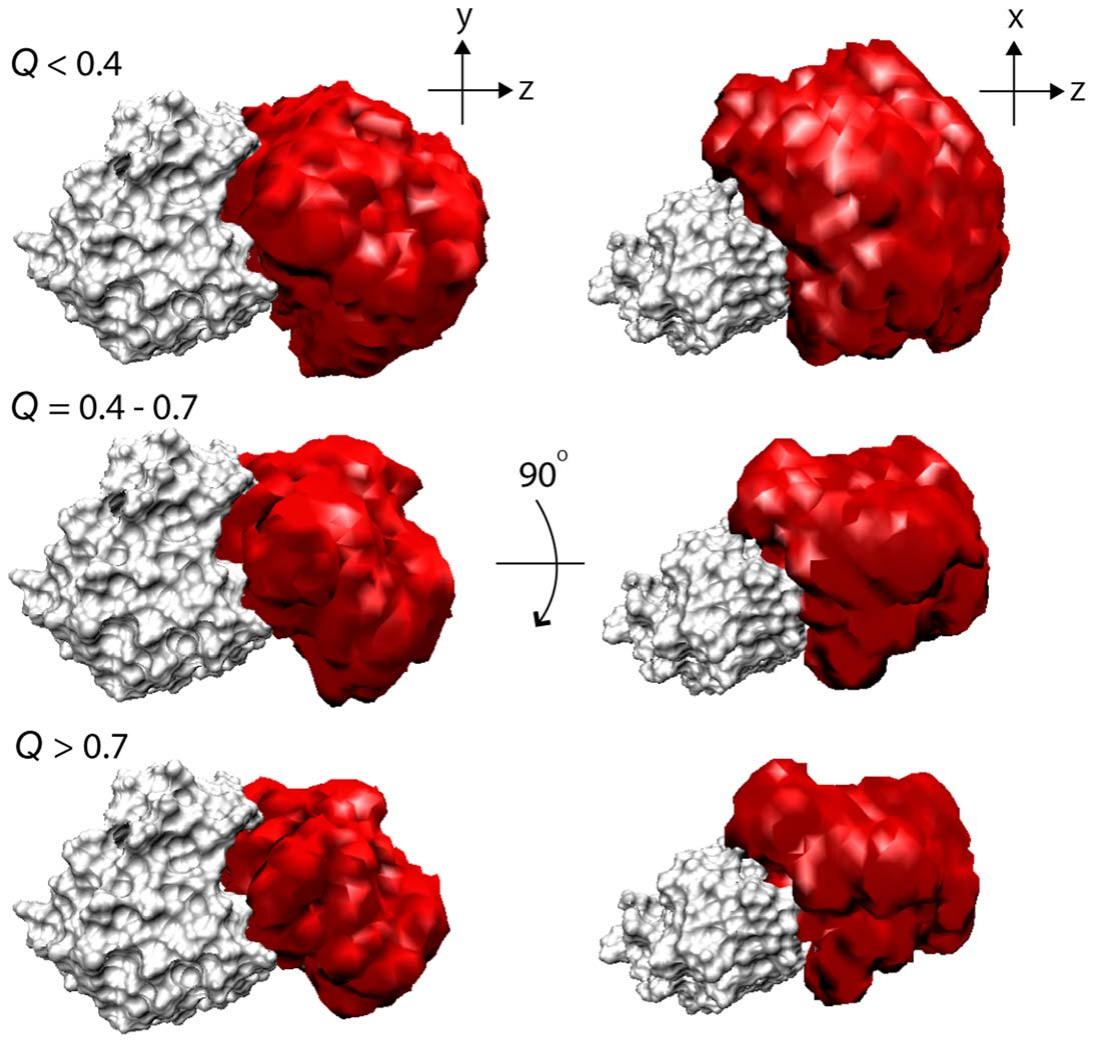
Narrowing of configurational space with increased *Q*. Occupancy maps of barstar C_α_ atoms with various *Q* ranges in unbiased replica of MSES simulation generated by VMD [70]. Three-dimensional grids are created using a bin width of 2 Å and the grid points occupied by C_α_ atoms in the unbiased MSES ensemble are shown in red. The coordinates are superimposed on the barnase molecule, which is shown in gray.

In Fig. 2, the process of the formation of inter-molecular interactions was illustrated in the distribution of the COM of barstar on the surface of barnase (along the *x-y* plane and the *x-z* plane; see the legend of Fig. 2 for the definition of the axes) for various ranges of *Q*, the fraction of the native inter-molecular contacts formed in the MSES ensemble (0≤*Q*≤1; the native contacts were defined as those having more than a 70% probability of occurrence in the MM simulation), as is used in the studies of protein folding. At a low *Q* range, barstar is positioned over a wide area on the surface of the barnase, where the distributions appear to largely spread in the *x*-direction compared to the *y*-direction. This is simply because there are two protrusions on barnase, one at Ser38 and the other at Glu60 and Gln104, which are respectively located above and below the barstar binding site along the *y*-axis, and this significantly restricts the barstar's motion (Figs. 2 and S3). The broad distribution for an increasing *Q*-value gradually converges to a more restricted area centering on the complex structure. The same distributions were also shown in the occupancy maps of the barstar molecule, representing its translational and rotational motions relative to barnase (Fig. 3); the space occupied by barstar is spread widely at *Q*<0.4 and becomes smaller with increasing *Q*. At *Q*>0.7, the space shrinks to the level in the MM simulation, going downhill to the bottom of the FES. This monotonous contraction of the distribution suggests that the FES of the barnase and barstar interactions is funnel-like downhill.

**Figure 4:**
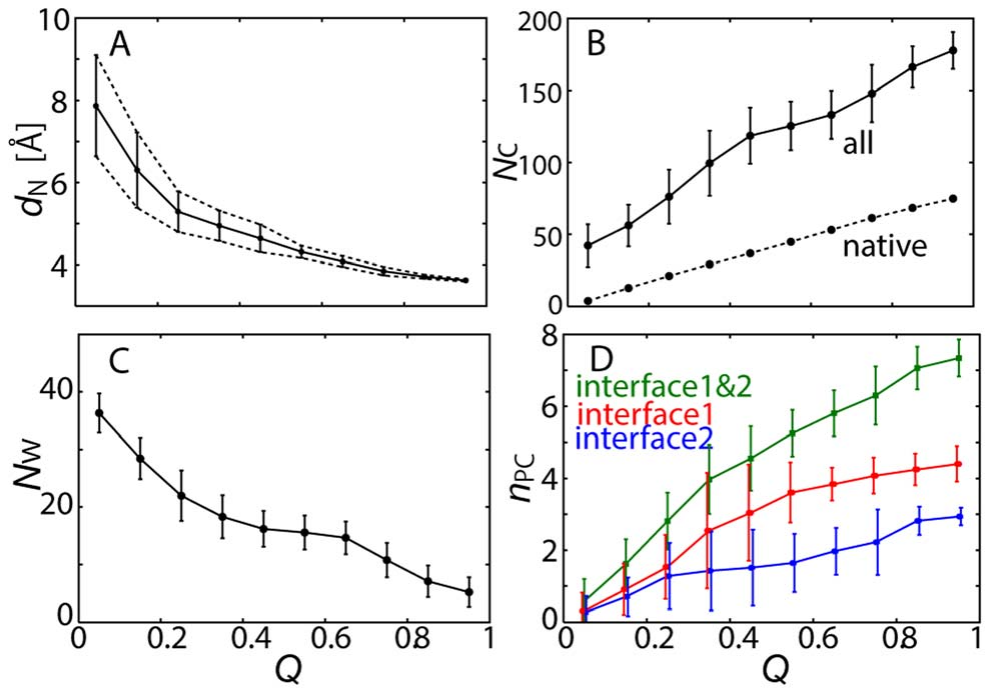
Free energy surfaces in terms of *Q* value. (A) Average inter-molecular distance calculated between native contacts, *d*_N_, which were given by average of inter-molecular distances for non-hydrogen atoms in simulation snapshots having specified *Q* value. (B) Number of inter-molecular contacts, *N*_C_, which are non-hydrogen atoms within 4 Å. Number of native contacts found in the native complex structure is also shown. (C) Number of hydrated waters at interface, *N*_W_, defined by oxygen atoms within 4 Å from interfacial non-hydrogen atoms. (D) Numbers of polar contacts for interfaces 1 and 2, respectively. The vertical lines are the standard deviations for each value.

**Figure 5:**
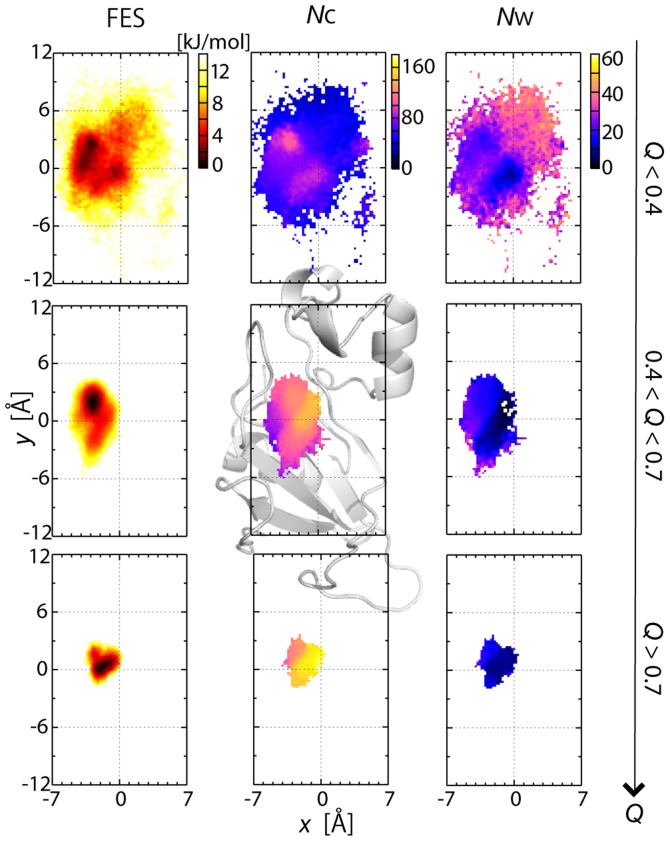
2-D Free energy surfaces in terms of *Q* value. 2-D free energy surfaces on *x-y* plane of probability distribution (FES), and numbers of contact atoms (*N*_C_) and hydrated waters (*N*_W_) are shown at depicted three *Q* ranges. The position is defined here as the center of mass of the interfacial atoms with inter-molecular contacts after superimposition to the crystal structure of barnase. The cartoon representation of barnase is also drawn for clarity in the *N*_C_ figure at 0.4<*Q*<0.7.

Using more quantitative statistics, we characterized the shape of the FES as a function of *Q*, i.e., the average distance between the native contacts (the inter-molecular contacts in the complex structure), *d*_N_, the number of inter-molecular contacts, *N*_C_ (two atoms within 4 Å), the amount of hydrated water at the interface, *N*_W_ (water within 4 Å from the protein interface), and the number of polar contacts, *N*_PC_ (out of the eight polar contacts listed in Table 1). In Fig. 4, we observed the association of the two proteins from the encounter forming the complex structure for the decrease of *d*_N_, which was accompanied by an increase in the number of protein-protein interactions (*N*_C_ and *N*_PC_) and a decrease in the amount of the hydrated water at the interface (*N*_W_). All of these values show gradual and smooth convergence to those of the complex structure with an increasing *Q*-value. The associated fluctuations, indicated by their standard deviations in the figure, also tend to converge to small values, implying a narrowing of the configurational space. The convergence of *N*_C_ and *N*_W_ with *Q* was also demonstrated on the *x-y* plane of the interface in Fig. 5: the two-dimensional energy landscape for the interfacial atoms again indicates the funnel-like downhill FES. The *N*_C_ and *N*_W_ distributions are complemental to each other; with an increased *Q* value, *N*_C_ increases and *N*_W_ decreases, indicating that the atom contacts are gradually formed and the solvents are excluded from the interface, yielding the complete complex structure.

All these data indicate the funnel-like downhill FES of the association process heading to the native complex structure: Various kinds of structural characteristics converge to those of the native complex structure as the *Q*-value increases, or more native contacts are formed. This is the same as the funnel picture of a protein folding whose ideally smooth funnel is expressed by the Go-model, in which the low-dimensional reaction coordinates, e.g., the native contacts, drive all the other reservoir variables to attain folding [55]–[57]. Similarly, in the association process after the encounter, the downhill FES or the funnel landscape was revealed.

**Figure 6:**
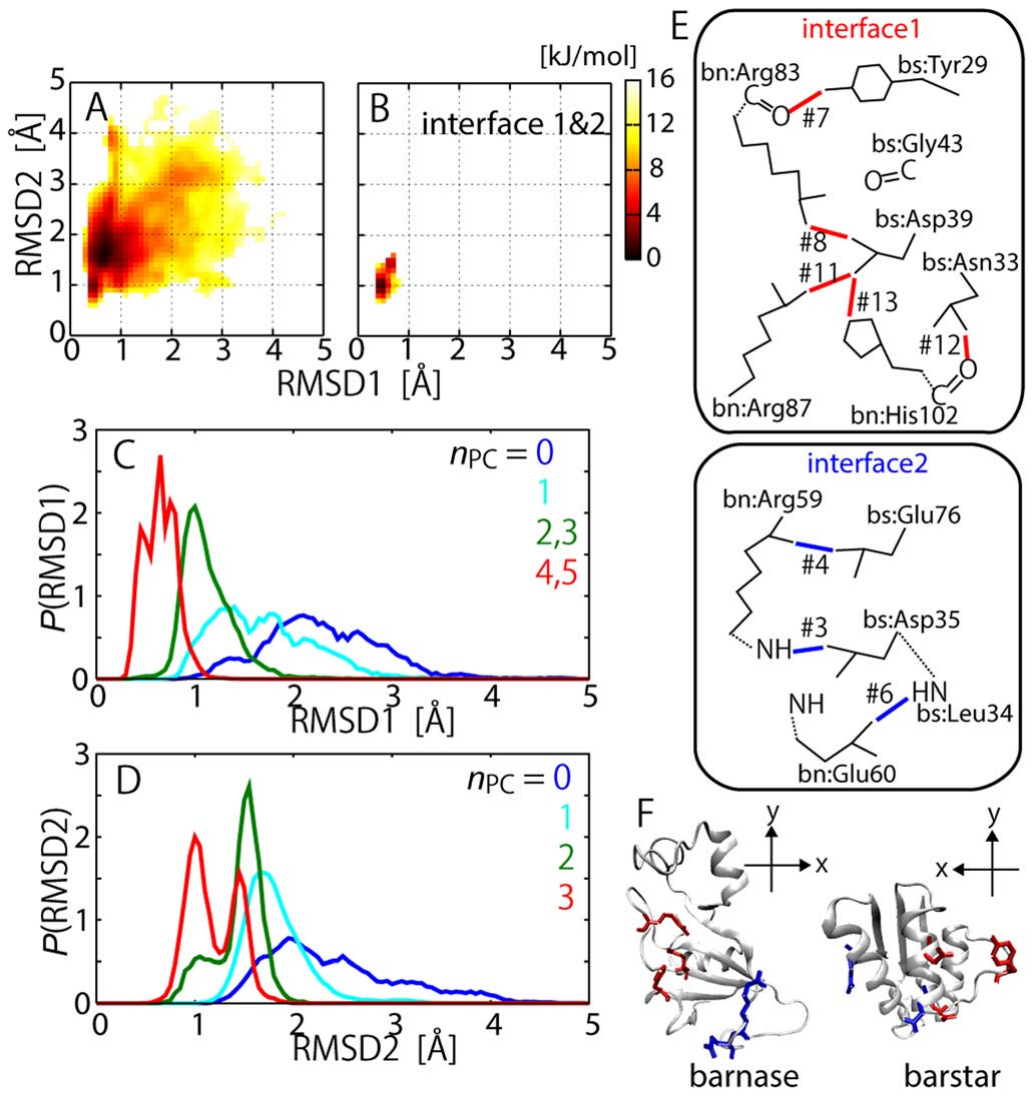
Formations of two localized interfaces. (A) 2D representation of FES along RMSD1 (non-hydrogen-atom RMSD for interface 1) and RMSD2 (non-hydrogen-atom RMSD for interface 2). In (B), the situation is the same but when both interfaces are formed. (C) and (D) show probability distributions along RMSD1 and RMSD2, respectively, when designated number of polar contacts are formed. (E) Native polar contacts at interfaces 1 and 2 (identifier is same as in Table 1). (F) Side-chain positions of interfaces 1 (red) and 2 (blue) of barnase and barstar.

**Figure 7:**
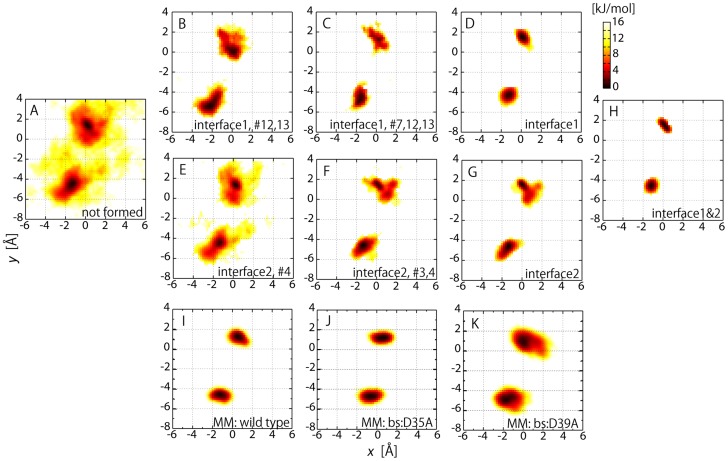
Free energy surfaces for two localized interfaces. 2-D free energy surfaces of barstar position on *x-y* plane of barnase: Two distributions are plotted on same figure for centers of mass of barstar residues comprising interface 1 (Tyr29, Asn33, and Asp39: upper right) and that for interface 2 (Asp35 and Glu76: lower left). In A–H, the distributions are drawn for the unbiased MSES ensemble under the respective conditions that the polar contacts given at the bottom (the identifier defined in Table 1) are formed. “Interface 1”, “interface 2”, and “interfaces 1&2” indicate the structures when all the polar contacts in interface 1 and/or 2 are formed, respectively. In I–K, the distributions obtained in the MM simulations starting from the complex structure are shown for the wild type (I) and the two mutants, bs:D35A (J) and bs:D39A (K).

We further focused on the FES of the more localized interactions of the inter-molecular polar contacts. The barnase-barstar interface was divided into two regions according to the geometric location of the interacting residues in the complex structure (see Table 1 and Fig. 6) [54]. The first group contains #7, 8, 11, 12 and 13, which form a network (“interface 1”) via relatively long side-chains (arginine, tyrosine, and so on) on the core of the interface, and the other consists of #3, 4 and 6, whose network (“interface 2”) is mostly via the main-chain atoms and located at the lower edge of the interface. Fig. 4D shows that each of these interfaces also exhibits funnel-like downhill FES. A more detailed picture is illustrated in Fig. 6A, in which the distribution of the interaction free energy expanded to two reaction coordinates, RMSD1 and RMSD2, i.e., the non-hydrogen atom RMSD's from the complex structure for interface 1 and for interface 2, respectively. Upon the formation of all the polar contacts on interfaces 1 and 2, the distribution converged to the restricted region of the complex structure (Fig. 6B). When further decomposing the two-dimensional plot into each of the one-dimensional distributions (Figs. 6C and 6D), we found that the increase in the number of native polar contacts in the interfaces progressively led to their native complex structures, respectively. These figures suggest that the inter-molecular interactions in the two localized interfaces appear to be formed almost independently along each funnel-like potential. This picture was confirmed in the projection of the probability distribution onto the *x-y* plane (Fig. 7): the positional fluctuations of the two interfaces are very large when no polar contacts are formed (Fig. 7A), while the interfaces are finally stabilized when all the contacts are formed (Fig. 7H). Figs. 7D and G demonstrate that the formation of interface 1 contributes more to the stability of the complex structure than that of interface 2. This may be, however, only due to the difference in the number of polar contacts, i.e., that the number of polar contacts at interface 1 (5) is larger than that of interface 2 (3). The MM simulations of the wild-type complex and two additional simulations of barstar mutants, D39A and D35A (reducing the number of polar contacts at interface 1 and interface 2, respectively), yielded consistent results with the MSES simulation results (Figs. 7I–K): the stability of D35A is comparable to that of the wild-type while D39A is much more destabilized than the wild type. This indicates a larger significance of Arg39 than Arg35 in the stabilization of the complex structure.

### Shape-complementarity-driven association

When looking at the detailed interactions on the two interfaces shown in Figs. 6, we noticed that these inter-molecular interactions were formed along a preferential pathway. As listed in Table 2, interface 1 was formed in the sequence, #12 or #13 (barnase(br):His102 – barstar(bs):Asn33 or Asp39)→#7 (br:Arg83 – bs:Tyr29)→#8 or #11 (br:Arg83 or Arg87 – bs:Asp39), and interface 2 has the sequence, #4 (br:Arg59 – bs:Glu76)→#3 (br:Arg59 – bs:Asp35)→#6 (br:Glu60 – bs:Leu34). These preferential pathways of the formation of the inter-molecular polar contacts are consistent with the FES in Figs. 7B–G, revealing that the two interfaces are more stabilized with the increasing number of formed polar contacts. The early stages of the association process predominantly involved the two residues in barnase, His102 on interface 1 and Arg59 on interface 2 (see Fig. S2 for the positions of the two residues). Since the configurational ensemble with *n*_PC_ = 1 in Table 2 does not correspond to sufficiently small *Q* values, i.e., <*Q*> = 0.21 at interface 1 and <*Q*> = 0.38 at interface 2, the polar contacts at the very beginning of the association process, *Q*<0.1, were further examined in Table 3. Just as in Table 2, we found that br:His102 with native contact #13 (br:His102 – bs:Asp39) and br:Arg59 with a non-native contact (br:Arg59N_η_ – bs:Asp35O_δ_; note that native contact #3 is between br:Arg59N and bs:Asp35O_δ_) are the most probable polar contacts at *Q*<0.1 (with the probability ≥0.2). The barstar counterparts of the polar contacts are Asp35 and Asp39 on helix 3 (residues 34–42), which is the helix most deeply interacting with the binding groove of barnase (see Fig. S2). Moreover, the molecular recognition between Arg59/His102 in barnase and Asp35/Asp39 in barstar has been considered to be crucial for molecular recognition in the barnase-barstar complex structure [47]–[50], [54], [58], [59]. This suggests that the inter-molecular interactions stabilizing the native complex structure are already formed at the very beginning of the association process after the encounter.

**Table 2:** Probability of occurrence of polar contacts, *p*, at interface 1 and interface 2, with various numbers of native contacts observed in MSES simulation^a^.

interface 1			*n*_PC_ = 1^b^	*n*_PC_ = 2	*n*_PC_ = 3	*n*_PC_ = 4
			*p* c			
7^d^	Arg83O	Tyr29O_η_	0.12	0.12	0.67	**0.87** e
8	Arg83N_η_	Asp39O_δ_	0.22	0.16	0.34	0.68
11	Arg87N_η_	Asp39O_δ_	0.00	0.01	0.09	0.55
12	His102O	Asn33N_δ_	0.31	**0.88**	**0.96**	**0.95**
13	His102N_ε2_	Asp39O_δ_	0.35	**0.85**	**0.94**	**0.98**

interface 2			*n*_PC_ = 1	*n*_PC_ = 2
			*p*	
3	Arg59N	Asp35O_δ_	0.02	**0.97**
4	Arg59N_η_	Glu76O_ε_	**0.98**	**0.97**
6	Glu60O_ε_	Leu34N	0.00	0.06

a Polar contacts formed between two atoms in either interface 1 or interface 2 are listed with probability of occurrence.

b *n*_PC_ is number of native polar contacts in Fig. 4E, for interface 1 (0≤*n*_PC_≤5) and interface 2 (0≤*n*_PC_≤3). In *n*_PC_ = 5 in interface 1 and *n*_PC_ = 3 in interface 2, the probability is unity by definition.

c Probability *p* has relation 
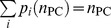
, where 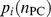 is probability for *i*-th identifier and *n*_PC_.

d Identifier is same as in Table 1.

e Bold numbers indicate probable polar contact with probability >0.8.

**Table 3:** Probability of occurrence of polar contacts, *P*, at interface 1 and interface 2, under condition *Q*<0.1 observed in MSES simulation^a^.

Interface 1			*P* b
8^c^	Arg83N_η_	Asp39O_δ_	0.05
7	Arg83O	Tyr29O_η_	0.11
	His102N_ε2_	Tyr29O	0.05
	His102N_ε2_	Asp35O_δ_	0.08
	His102N_ε2_	Asp35O	0.03
13	His102N_ε2_	Asp39O_δ_	**0.20** d
12	His102O	Asn33N_δ_	0.06

Interface 2			
	Arg59N_η_	Asp35O_δ_	**0.34**
4	Arg59N_η_	Glu76O_ε_	0.12

a Polar contacts formed between two atoms in either interface 1 or interface 2 are listed with probability of occurrence when fraction of native contact, *Q*<0.1. All the contacts with the probability >0.03, including the non-native contacts, are listed here.

b Probability was calculated as (# of snapshots in MSES ensemble having polar contact and *Q*<0.1)/(# of snapshots in MSES ensemble satisfying *Q*<0.1).

c Identifier is same as in Table 1.

d Bold numbers indicate probable polar contact with probability ≥0.2.

**Figure 8:**
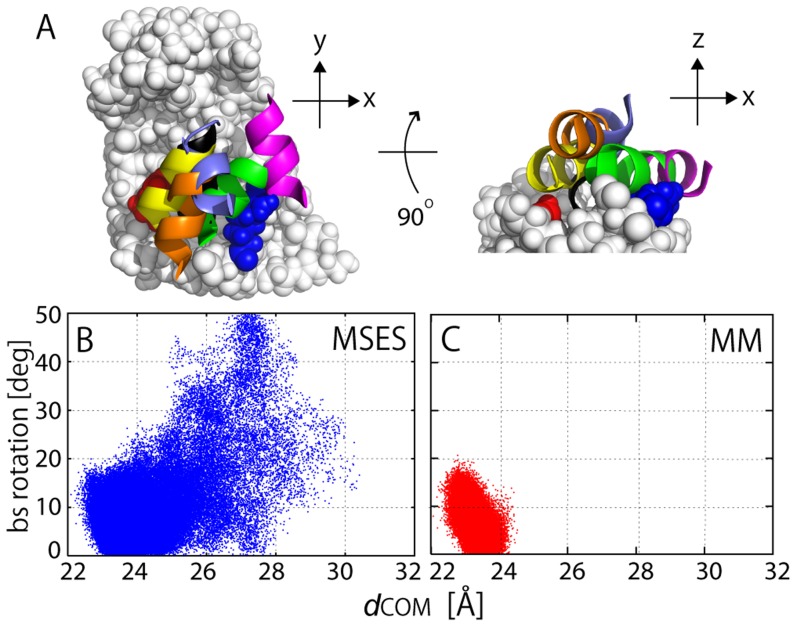
Downhill FES via shape complementarity. (A) Five snapshots of helix 3 (residues 34–42) of barstar for *Q*<0.1, together with helix 3 in native complex structure (black; in view from bottom). The white space filing model is barnase in which Arg59 (blue) and His102 (red) are colored in the figure. (B) and (C) 2D representation of FES along COM distance, *d*_COM_, and rigid-body rotation angle of barstar from native complex structure, defined by change in direction of vector connecting Cα atoms of Asn33 and Asp83 between snapshot and native complex structure (see Fig. S3): (B) MSES simulation and (C) MM simulation.

A clue for understanding these native contacts formed in the early stages of association was found in the structural information for the transition state derived from the kinetic experiments by Schreiber *et al.* [57]. Schreiber *et al.* found that the transition-state structures had the binding surfaces of the two molecules correctly aligned as in the native complex structure. We found a similar feature in the early stages of the association process in the MSES simulation. At *Q*<0.1, the barstar orientation is already restricted near the native alignment, although the barstar helix is “floating” above the binding surface of barnase (Fig. 8A, showing only helix 3 for clarity). The later stages of the association process, including all the *Q*-ranges, have a similar distribution of the orientation of barstar (Fig. 8B); the orientation angle of barstar is within ∼50 deg, although this is a much wider range than that of the MM simulation (Fig. 8C). The helix 3 of barstar appears to preferentially make contacts with a neighboring residue on barnase, either Arg59 or His102, depending on its position on the binding surface (see Table 3). It is thus understood that the native-like polar contacts in the very early stages of the association process occur due to the near-native orientations of barstar.

The restriction of the barnase/barstar orientation can be attributed to the extensive shape complementarity between the two molecules (see Fig. S3). The shape complementarity between concave barnase and convex barstar mainly comes from the protrusions at Ser38, Glu60, and Gln104 forming the binding site of barnase and strictly precludes barstar's motion. We found in the MSES simulation that the steric hindrance was frequently seen in the residue pairs, br:Ser38-bs:Tyr29, br:Glu60-bs:Trp38 and br:Gln104-bs:Asp39 (see Fig. S3); on barstar the interfacial residues with large side-chains appear in the collision. In principle, barstar would make a full rotation when it is fully separated from barnase. However, the MSES simulation sampled up to the rotation angle of 50 deg, and the range of *d*_COM_ = ∼30 Å (Fig. 8B), and maintained *N*_C_>∼40 (Fig. 4B). Beyond this range, the two molecules are completely separated (*N*_C_ = 0), and cause energetically unfavorable states that were not easily sampled even by the MSES simulation.

As an extrapolation of the landscape obtained above, we conducted a simple simulation in which the relative motion of the two molecules was restricted only to the rigid-body translation and rotation along the COM axis. The result shows that the accessible rotation angle decreased drastically when *d*_COM_<27 Å and atomic clashes impeded the free rotation of barnase and barstar (Fig. S4). It means that strong geometrical complementarity of the complex structure already occurs at the COM distance of ∼5 Å away from the crystal structure whose *d*_COM_ = 22.3 Å. The geometrical complementarity is also seen in the sudden increase in *N*_C_ at *d*_COM_<26 Å. Note that the configurational space thus derived is very limited and different from the results in the MSES simulation including all degrees of freedom. However, this simple simulation may demonstrate the extensive influences from the shape complementarity to the energy landscape.

## Discussion

We have successfully simulated the association-dissociation processes of the barnase-barstar complex in atomic detail including explicit solvent by use of multiscale enhanced sampling. The following scenario of the association process of the barnase-barstar system can then be considered based on the above observations. In the encounter complex, the electrostatic complementarity determines the interacting surface (Fig. S3), and barstar retains rotational freedom in the encounter complex [20], [23], [27], [52]. Once barstar approaches barnase closer than *d*_COM_ = ∼30 Å, or goes beyond the transition state, barstar is caught in the binding pocket of barnase and thus loses the rotational freedom due to the extensive shape complementarity. This native-like orientation allows the interface residues to make inter-molecular contacts, including the native and near-native contacts. The formation of these contacts successively induces the inter-molecular interactions listed in Table 1 to produce the downhill funnel-like landscape, yielding the final complex structure. The diffusion limited rate constant of the association process [51] can be attributed to this funnel-like landscape.

Such an extremely smooth downhill landscape may be found exclusively in a barnase-barstar system exhibiting extraordinarily strong interactions and fast association kinetics [50], [51]. This smooth landscape in the protein-protein interaction may correspond to the landscapes of the fast folding of small proteins, which also has smooth downhill landscapes [55]–[57], [60], [61]. Another class of protein complex systems with a lower affinity should have a more rugged landscape, as in the folding of larger proteins. The MSES simulation has opened up the possibility to delineate much more complex landscapes in the protein-protein interactions.

## Methods

### MSES simulation

The MSES simulation is described in detail in the literature [29]–[31]. We provide a brief summary here. This introduces a multiscale system in which both an all-atom system, composed of protein molecules and surrounding solvents (MM; **r**_MM_), and the associated coarse-grained system (CG; **r**_CG_) are simulated in the following method. Since a multiple CG method was used in this study, we describe the method using multiple CG systems [31]. The Hamiltonian, *H*, for the MSES simulation is given by
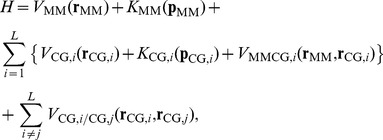
(1)with
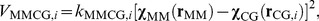
(2)where *V*_MM_ (*K*_MM_) and *V*_CG_ (*K*_CG,*i*_) are the potential (kinetic) energy functions for MM and the *i*-th CG (*i* = 1, 2,…, *L*; *L* is the number of CG models), respectively, and the number of degrees of freedom in each CG, *M*, is much smaller than that of MM, *N*. The CG models can be arbitrarily chosen according to prior knowledge or experimental information. In this study, a C_α_ model of barnase and barstar (*M* = 199 atoms×3) was used with *L* = 2. The term, *V*_MMCG,*i*_, defines the coupling (harmonic constraint) for *K* variables, **χ**_CG_, determined by CG coordinates, with the force constants *k*_MMCG,1_ = *k*_MMCG,2_≡*k*_MMCG_, to drive the MM system by the accelerated dynamics of the two CG systems, where the *K*-dimensional vector **χ**(**r**_MM_) is a projection of **r**_MM_ onto the *K*-dimensional space. Here, a set of *K* inter-molecular C_α_ distances between barnase and barstar was used as the variables **χ**_MM_ and **χ**_CG_ in *V*_MMCG,*i*_ (*K* = 104 was used in this study for Cα atom pairs with pairwise distances less than 10 Å in the crystal structure of the complex, and therefore *K*<*M*≪*N∼*10^5^). The details of the MM and CG parameters are given in the next section. The potential *V*_CG,*i*/CG,*j*_ produces repulsive force between a pair of the CG systems to avoid the overlap of the CG systems and then to maintain the sampling efficiency. Here, the following function was used [31];
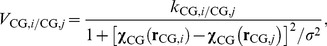
(3)where *k*_CG,*i*/CG,*j*_ is a coupling constant and *σ* is a parameter to determine the correlation distance.

The ultimate goal of the simulation is to derive the free energy surface solely from *V*_MM_ without any bias due to the coupling *V*_MMCG_. Therefore, it is necessary to eliminate the coupling influence, or to extrapolate the system to the one with *k*_MMCG_ = 0. For this purpose, the Hamiltonian replica exchange method [62], [63] is adopted, in which many replicated systems are assigned various values of *k*_MMCG_, from a large value to zero. The exchange probability between replicas *m* and *n*, satisfying the detailed balance condition, is given by
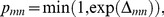
(4)with
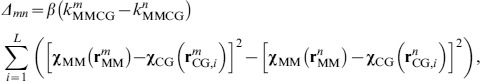
(5)where *β* is the inverse temperature of the MM-CG coupled system. Eq. 3 indicates that the probability is determined by the difference between **χ**_MM_(**r**_MM_) and **χ**_CG_(**r**_CG,*i*_) defined in a small dimension (*K*). Because of *K*≪*N*, Δ*_mn_* can be kept small enough to provide a high exchange probability *p_mn_* irrespective of the size of the system *N*. This guarantees an excellent scalability highly superior to that of the conventional temperature replica exchange method, where the difference in the potential energy of MM (scaling up with *N* ^2^) determines the exchange probability Δ*_mn_*.

### Potential energy functions and kinetic parameters for MSES

The energy functions, *V*_MM_+*K*_MM_, *V*_CG,*i*_+*K*_CG,*i*_ and the coupling term in Eq. 1 were defined as follows. For the all-atom potential energy *V*_MM_, AMBER ff99SBildn was used [64]. The CG potential *V*_CG_ was prepared as the sum of two terms representing the intra-molecular interactions (*V*_CG,intra_) and the inter-molecular interactions (*V*_CG,inter_). For *V*_CG,intra_ the potential function of the C_α_ elastic network model was used [65]. The force constant and the cut-off length in the elastic network model were set at 1.8 kcal/mol/Å^2^ and 12 Å, respectively. For *V*_CG,inter_ the Lennard-Jones potential with a potential depth of 0.2 kcal/mol and a soft (harmonic) boundary with a force constant of 5 kcal/mol/Å^2^ at 10 Å apart from the minimum of the LJ potential was applied to the selected 104 C_α_ atom pairs. The 104 pairs were selected as those of the interfacial residues under the condition of a C_α_ atom distance less than 10 Å in the crystal structure of the complex (PDB: 1BRS [49]). The LJ potential is used for the attraction between the two CG models, and the soft boundary potential is to avoid a too large separation. The mass of the CG model was set as *m*_CG_ = 10,000.

### Computations

The starting structure was taken from the X-ray structures in the PDB entry 1BRS [40], in which Cys40 and Cys82 were mutated to Ala [48], [49]. We used the C40/82A mutant for the simulations. Rectangular simulation box was constructed with a margin of 12 Å to the boundary of the simulation box, resulting in the dimension, 73.8 Å×71.8 Å×83.8 Å. The solution system contained ∼11,000 TIP3P water molecules [66] together with four sodium ions to neutralize the simulation system. There were a total of 35,656 atoms in the system. The MSES simulations were performed using the class library for multicopy and multiscale MD simulations. The MM simulations were under constant temperature and pressure (NPT) conditions at *T* = 300 K and *P* = 1 atm using Berendsen's thermostat and barostat [67] at a relaxation time of 1 ps, and using the particle mesh Ewald method [68] for the electrostatic interactions. The simulation time step (*dt*) was 2 fs using constraining bonds involving hydrogen atoms via the SHAKE algorithm [69]. The CG simulation was also performed by using a Berendsen's thermostat under a constant temperature (NVT) condition of *T* = 300 K with *dt* = 2 fs. The parameters, *k*_CG1/CG2_ and σ^2^ in Eq. 2, were set at 15 kcal/mol and 10 Å^2^, respectively. For the MSES simulations, 12 replicas were used with *k*_MMCG_≡*k*_MMCG1_ = *k*_MMCG2_ = 0, 0.001, 0.0024, 0.0046, 0.0084, 0.015, 0.022, 0.03, 0.042, 0.056, 0.072 and 0.09 kcal/mol/Å^2^. The replica exchange was attempted every 20 ps. The total simulation time of MSES was 100 ns, extending 12×100 ns = 1.2 µs simulation time. The convergence of the simulation was confirmed by the *d*_COM_ distribution, which was calculated using several partial trajectories (Fig. S5). For comparison, the conventional equilibrium MD (MM simulation) was also performed starting at the complex structure during the same simulation time (i.e., 100 ns). The MM simulations of the wild-type and the two mutants, bs:D35A (PDB: 1X1Y) and bs:D39A (PDB: 2ZA4) [54], were also conducted under the same simulation conditions as that described above.

## Supporting Information

Figure S1 Time course of *V*_MMCG_ for all the 12 model replicas. Model replica indicates the replica fixed not by *k*_MMCG_, but by the configuration.
(TIF)

Figure S2 Free energy profile along *d*_COM_: coarse-grained simulation (CG), MSES simulation accumulated ensemble for all replicas (MSES, all), and unbiased ensemble derived from MSES simulation (MSES).
(TIF)

Figure S3 (A) Electrostatic potential surfaces of barnase and barstar interfaces generated by using APBS plugin with charge smoothing of PyMOL. The positive and negative charges are drawn in blue and red, respectively. Cartoon representations of the barnase and barstar interfaces and the complex structure are shown in (B) and (C), respectively. The residues are labeled and helix 3 in barstar (bs:34–42) is shown in red, which are essential in the association process. The direction of *x*-axis, a vector connecting C_α_ atoms of Asn33 and Asp83, is also shown as a dashed line in (C).
(TIF)

Figure S4 Accessible rotation angle of the crystal complex structure as function of center-of-mass (COM) distance (*d*_COM_) is shown by blue curve. This was calculated simply as the range of possible rotation angle of the rigid-body barnase and barstar molecules around the COM axis, i.e., when the COM's were separated by *d*_COM_ along the COM axis, barstar was rotated against barnase around the COM axis before a van der Waals atom clash occurred. The rotation was started from the crystal structure. The number of inter-molecular atom contacts, *N*_C_, at the crystal structure translated by *d*_COM_ along the COM axis is also represented by red curve.
(TIF)

Figure S5 The distributions of *d*_COM_ using the whole (0–100 ns) and the three parts (0–70 ns, 0–80 ns and 0–90 ns) of the trajectories are shown by red, blue, green, and magenta, respectively.
(TIF)
