# Supplementary figures and images for: Energy Landscape of All-Atom Protein-Protein Interactions Revealed by Multiscale Enhanced Sampling
Source: PLoS Comput Biol. 2014 Oct 23;10(10):e1003901. doi: 10.1371/journal.pcbi.1003901 (PMC4207830; doi:10.1371/journal.pcbi.1003901)

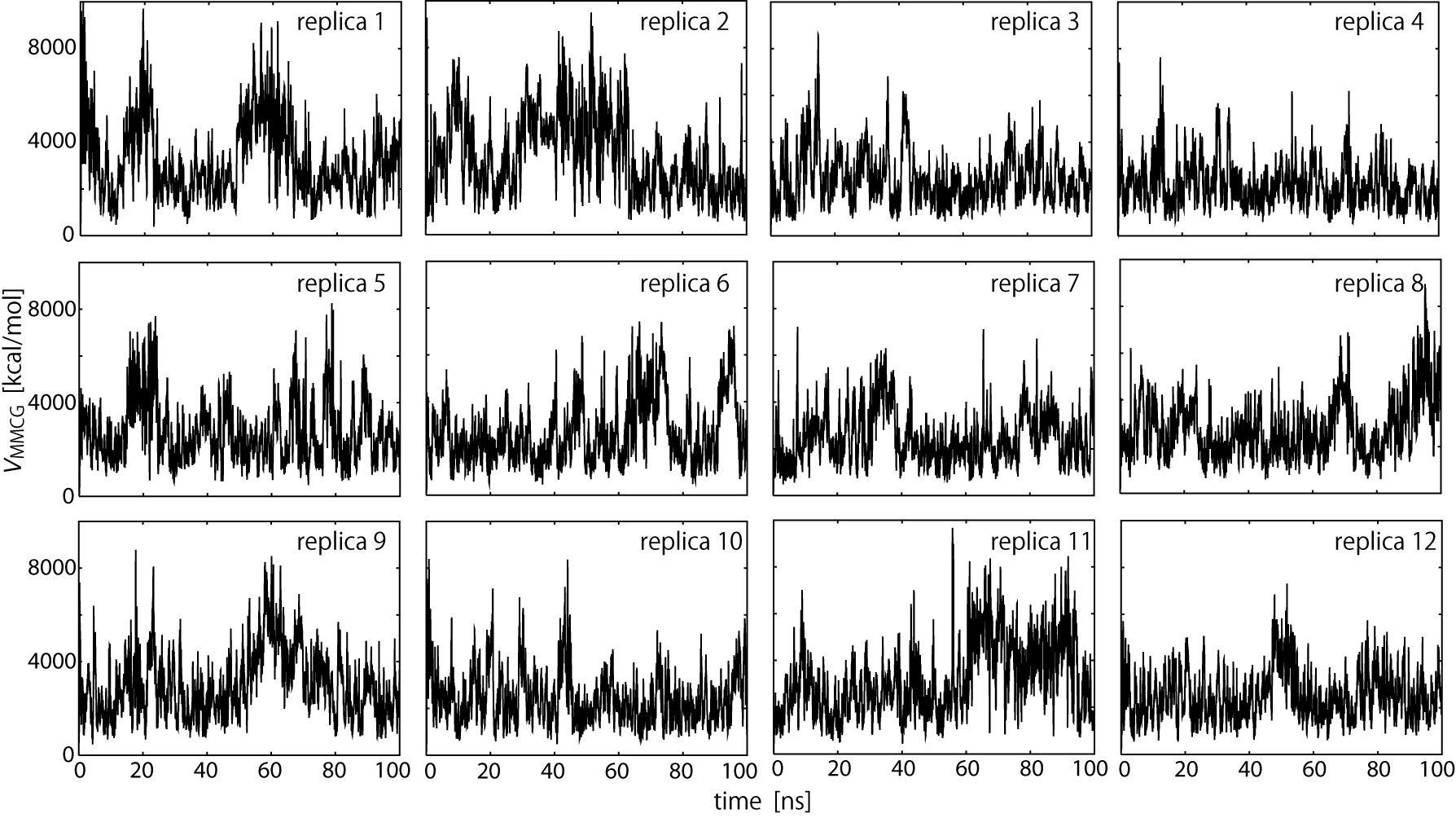

Supplement: Figure S1 — Time course of V MMCG for all the 12 model replicas. Model replica indicates the replica fixed not by k MMCG, but by the configuration. (TIF) [file pcbi.1003901.s001.tif]

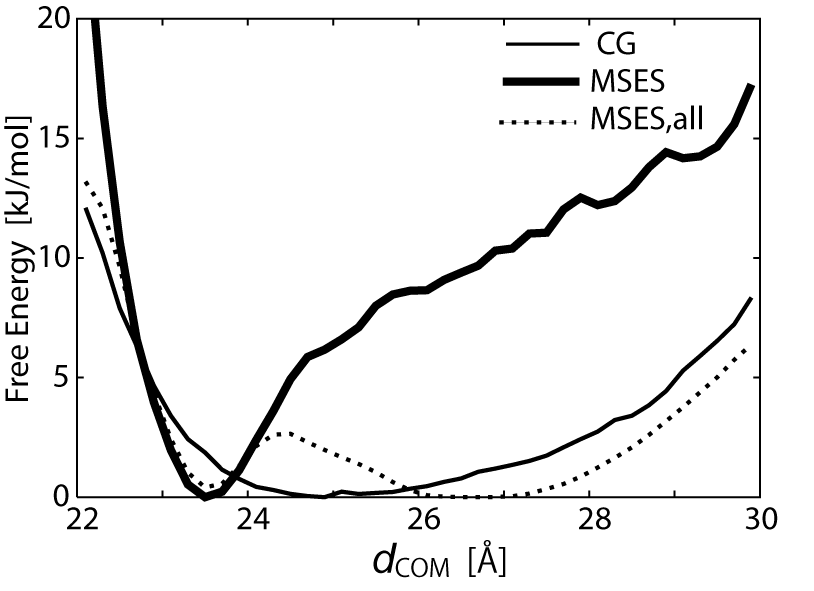

Supplement: Figure S2 — Free energy profile along d COM: coarse-grained simulation (CG), MSES simulation accumulated ensemble for all replicas (MSES, all), and unbiased ensemble derived from MSES simulation (MSES). (TIF) [file pcbi.1003901.s002.tif]

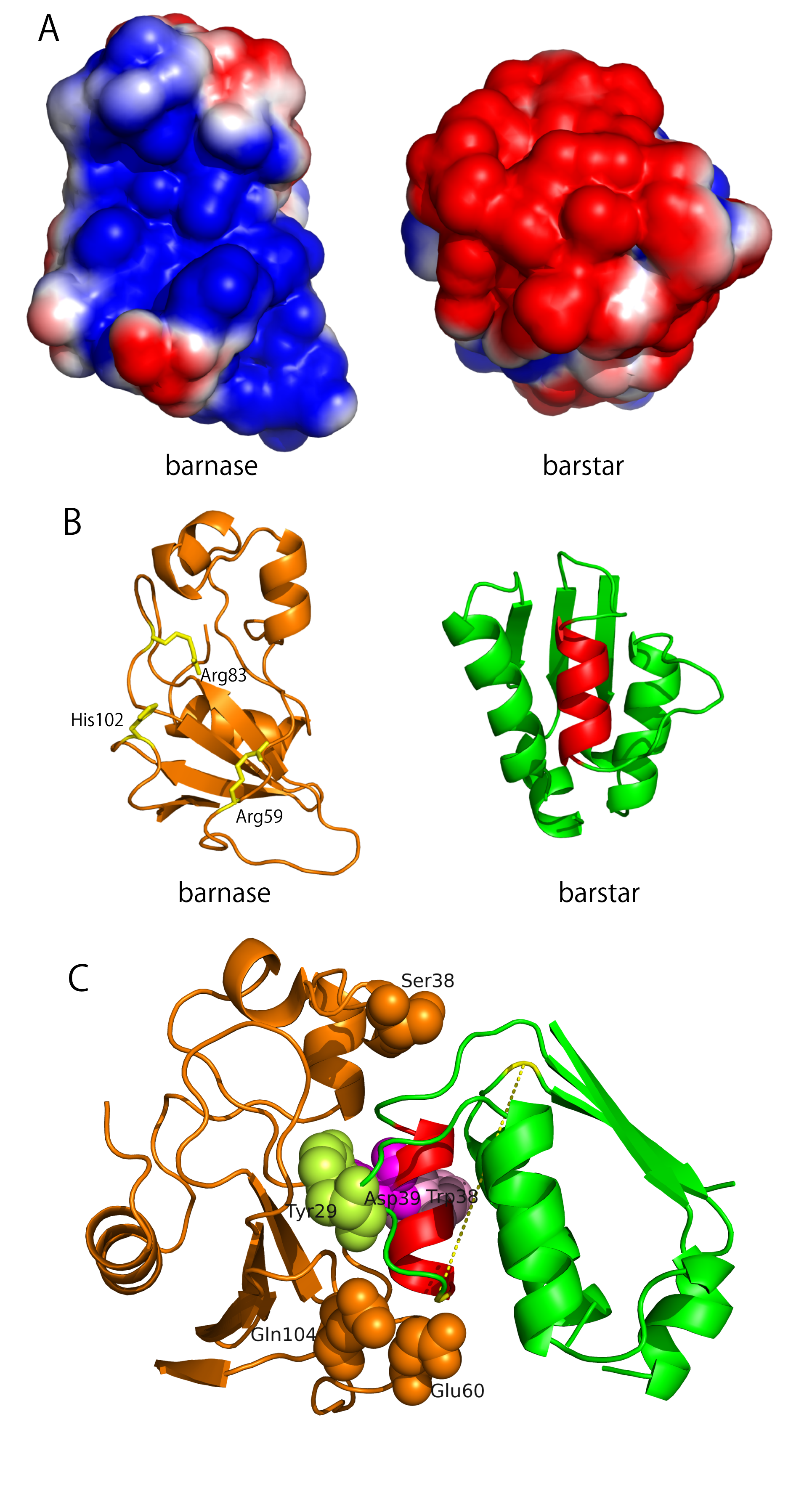

Supplement: Figure S3 — (A) Electrostatic potential surfaces of barnase and barstar interfaces generated by using APBS plugin with charge smoothing of PyMOL. The positive and negative charges are drawn in blue and red, respectively. Cartoon representations of the barnase and barstar interfaces and the complex structure are shown in (B) and (C), respectively. The residues are labeled and helix 3 in barstar (bs:34–42) is shown in red, which are essential in the association process. The direction of x-axis, a vector connecting Cα atoms of Asn33 and Asp83, is also shown as a dashed line in (C). (TIF) [file pcbi.1003901.s003.tif]

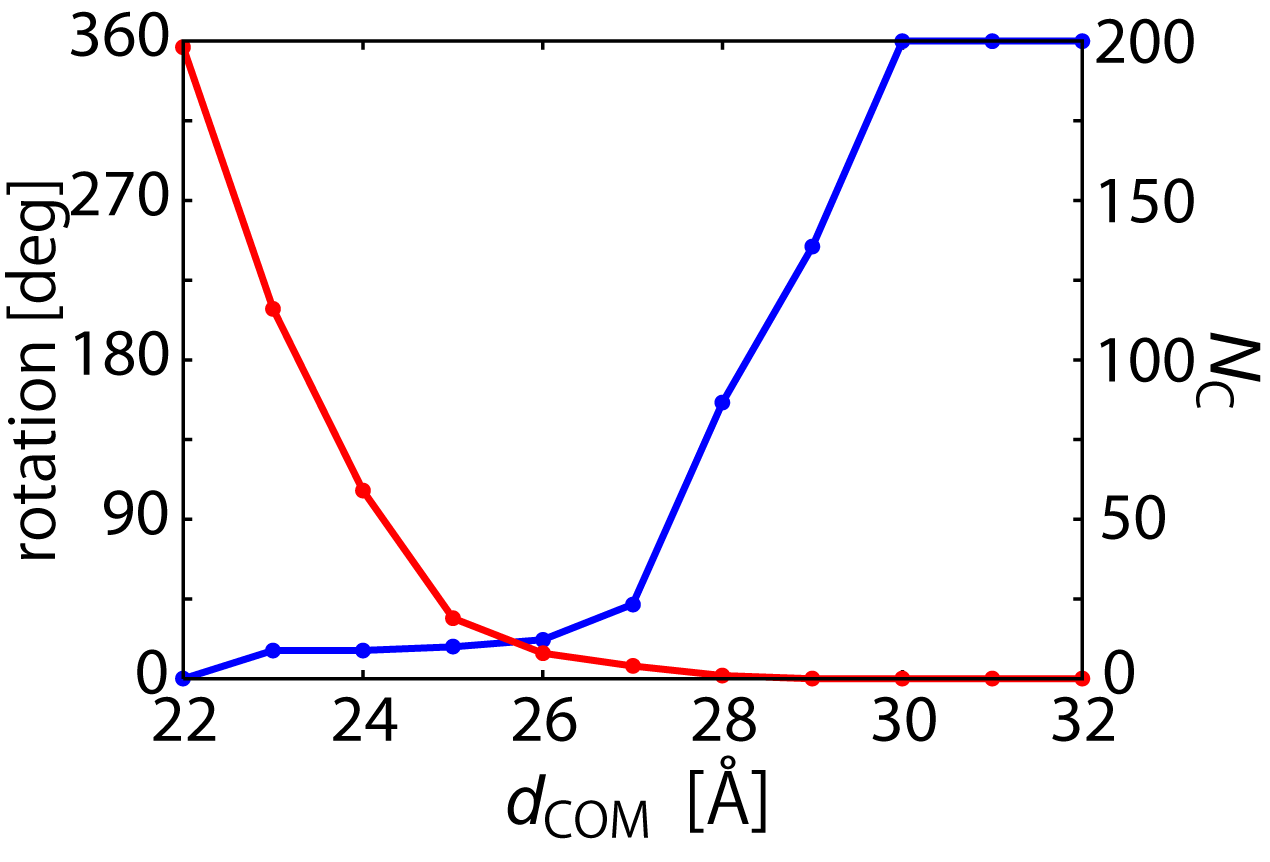

Supplement: Figure S4 — Accessible rotation angle of the crystal complex structure as function of center-of-mass (COM) distance (d COM) is shown by blue curve. This was calculated simply as the range of possible rotation angle of the rigid-body barnase and barstar molecules around the COM axis, i.e., when the COM's were separated by d COM along the COM axis, barstar was rotated against barnase around the COM axis before a van der Waals atom clash occurred. The rotation was started from the crystal structure. The number of inter-molecular atom contacts, N C, at the crystal structure translated by d COM along the COM axis is also represented by red curve. (TIF) [file pcbi.1003901.s004.tif]

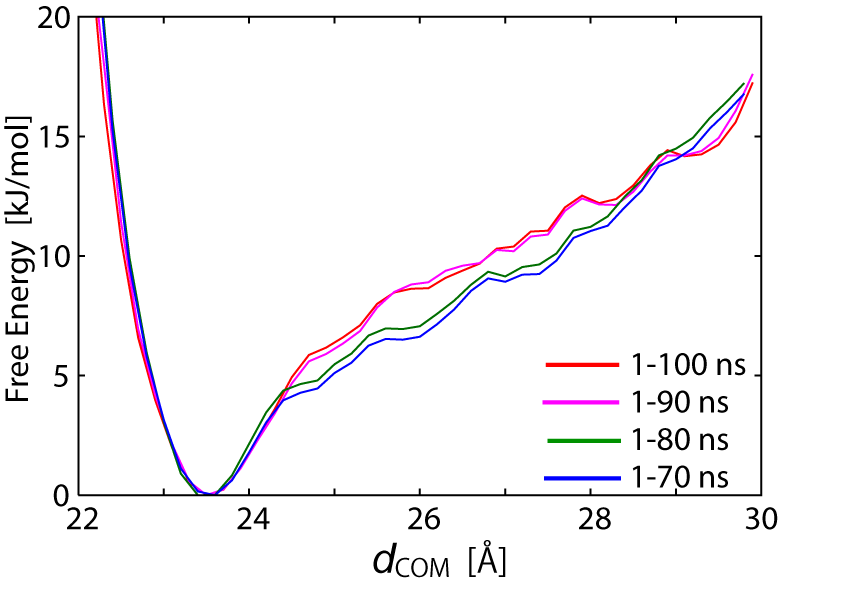

Supplement: Figure S5 — The distributions of d COM using the whole (0–100 ns) and the three parts (0–70 ns, 0–80 ns and 0–90 ns) of the trajectories are shown by red, blue, green, and magenta, respectively. (TIF) [file pcbi.1003901.s005.tif]
